# Morphological Variability of the Thigh Muscle Traps in an Ultrasound That Awaits Clinicians

**DOI:** 10.3390/jcm14020464

**Published:** 2025-01-13

**Authors:** Marta Pośnik, Nicol Zielinska, Adrian Okoń, Andrzej Węgiel, Mariola Głowacka, Łukasz Olewnik

**Affiliations:** 1 Department of Radiology, Diagnostic Imaging and Interventional Radiology, Medical University of Lodz, 90-419 Lodz, Poland; marta.posnik@stud.umed.lodz.pl; 2 Department of Clinical Anatomy, Masovian Academy in Płock, 09-402 Płock, Poland; nicol.zielinska@stud.umed.lodz.pl (N.Z.); adrian.okon@umed.lodz.pl (A.O.); 3 Department of Rheumatology, Medical University of Lodz, 90-549 Lodz, Poland; andrzej.wegiel@stud.umed.lodz.pl; 4 Nursing Department, Masovian Academy in Płock, 09-402 Płock, Poland; m.glowacka@mazowiecka.edu.pl

**Keywords:** ultrasound, imaging study, morphological variations, sartorius, tensor of the vastus intermedius, quadriceps femoris, adductor muscles, tensor fasciae suralis, semitendinosus, semimembranosus, biceps femoris

## Abstract

**Objectives:** Muscles and their tendons present a considerable diversity of morphological variations. The aim of this study was to explore variants of muscles and tendons from compartments of the thigh and to raise awareness about potential problems during ultrasound examination. **Materials and Methods:** This comprehensive review of the literature was created on the basis of scientific articles sourced from PubMed. The search included all relevant papers related to the topic, ensuring that the most up-to-date studies were incorporated. In order to achieve these results, we created the exclusion criteria and extracted papers that did not meet the requirements of our review. Relevant papers were incorporated, and tracking of citations was fulfilled. The described method allowed for a broad yet detailed understanding, ensuring that the review of the literature covers all key aspects of the presented research. **Results:** Various aspects of thigh muscle anomalies were already undertaken; however, as this study has shown, current knowledge, while valuable, is insufficient to draw definitive conclusions regarding the prevalence and clinical implications of these muscle variations. A more robust body of ultrasound-based research is essential to accurately characterize these anomalies, establish their frequency, and assess their impact on clinical decision-making, including diagnostic accuracy, surgical planning, and therapeutic interventions. **Conclusions:** Numerous anatomical variations of the thigh muscles and tendons that were described in literature over the years might have clinical implications and could lead to mistakes during diagnosis by ultrasound imaging.

## 1. Introduction

Ultrasound imaging is a facility that clinicians from all around the world are currently turning to due to its increasing availability and lower costs of evaluation compared to other imaging devices. However, the images obtained during this examination may exhibit variability, with anatomical variations likely being one of the contributing factors.

It is well known that the human body continues to evolve, and the morphological variability of its structures is an undeniable effect of such a course of events [1]. Even though vessels, arteries, and nerves are proven to be more variable than muscles and their tendons, they still present numerous variants in a number of additional components: heads, slips or bands, accessory muscles, a complete absence of well-known structures, or unusual origins/sites of insertion [2].

The aim of this study was to explore the variations of thigh muscles found in scientific literature and to consider the possible effects of their presence during ultrasound examination. Muscular components can be easily mistaken for other soft-tissue structures if one does not expect those in certain locations. So, in order to prevent misdiagnosis and the wrong path of treatment, it is important to enhance awareness of these.

## 2. Methods and Review Design

In order to find suitable publications for the presented review of the literature, an electronic search in the “PubMed” database was performed.

The exclusion criteria included the following:

- Manuscripts written in any language other than English.
- Lack of information regarding the description of the typical anatomy/morphological variability in a structure covered by the topic of this review/clinical significance/application of imaging studies/the use of US in diagnosis.
- Articles focused mainly on methods of visualization other than US imaging.
- Type of the article, including expert opinion/letter to the editor/conference report.
- Publication after July 2024.

From each of the selected literature positions, we extracted the information from each article consistent with the subjects covered in the presented review. In total, 79 positions were included. In order to identify the included publications, we used a citation tracking system (Mendeley Reference Manager).

**Figure 1:**
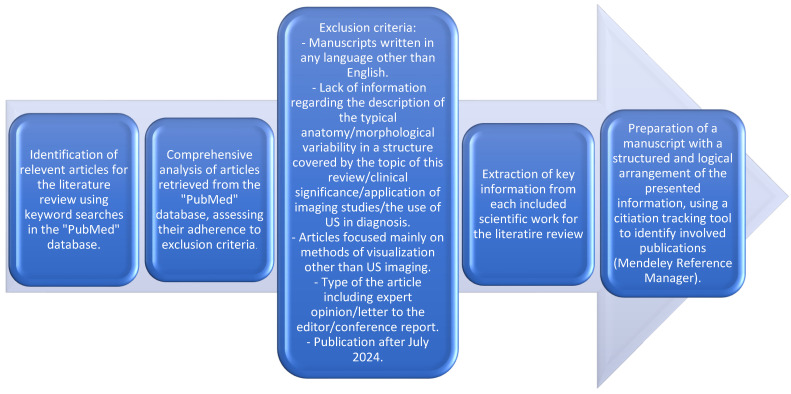
Schematic summary of the method used during preparation of the presented review of the literature.

Each muscle description was assigned according to the anatomical division of the thigh muscles, and each depiction presents information on each muscle in the following order: typical anatomy, variable morphology, and considerations on its clinical meaning and occurrence in ultrasound studies, depending on the available literature on the subject (Figure 1).

## 3. Discussion

### 3.1. Anterior Thigh

The anterior thigh is typically composed of the sartorius muscle (SM) and components of the quadriceps femoris (QF): Rectus Femoris (RF), Vastus Medialis (VM), Vastus Lateralis (VL), and Vastus Intermedius (VI).

### 3.2. Sartorius Muscle

The SM is typically described as a superficial muscle located in the anterior compartment that originates from the anterior superior iliac spine and the notch between the anterior superior and anterior inferior iliac spine that runs down through the thigh and inserts onto the proximal end of the tibia right below the medial condyle by means of the pes anserinus (PA).

**Figure 2:**
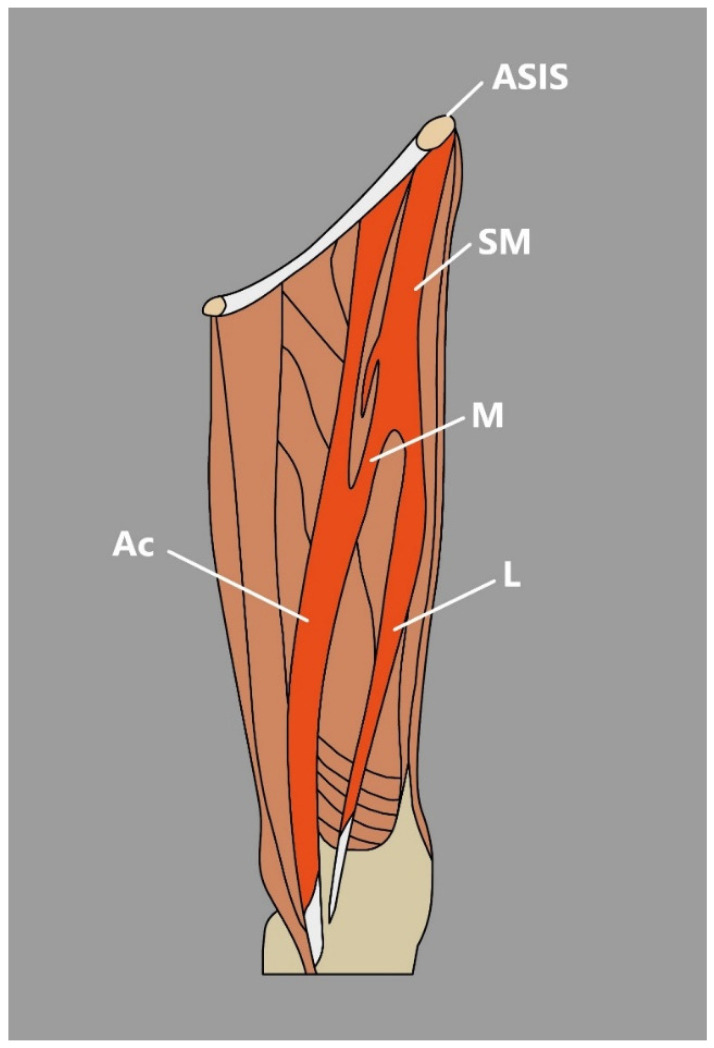
Schematic representation of the variable sartorius muscle reported by Kim et al. [7]. ASIS: anterior superior iliac spine. SM: sartorius muscle. L: lateral head of the sartorius muscle. M: medial head of the sartorius muscle. Ac: accessory sartorius muscle.

Even though the available literature exploring SM morphology is not considered detailed, several anatomical variations have been noted:

- The SM might be observed as a cone-shaped or rectangular structure [3]. Occasionally, an extra tendon that starts muscle duplication in the form of a hollow occurs, sometimes partial duplication or the distal muscular fragment can be noted, or discontinuous SM that consists of three muscular parts and two connecting tendinous parts [3].
- Tsakotos et al. [4] reported a case of a bicaudatus SM that was divided into anterior and posterior parts on its distal third part of the muscle. Additional tendons that attach distally to the anteromedial aspect of the patella, medial condyle, or femur can be occasionally spotted [5].
- There are also reports about accessory heads of origin that usually arise from below the anterior superior iliac spine, pubic bone, iliopectineal line, or inguinal ligament [5]. Natsis et al. [6] reported a case of biceps-bicaudatus SM, where both heads (lateral and medial) originated from the anterior superior iliac spine and where the lateral head was additionally split into a lateral and medial bundle.
- Kim et al. [7] Presented an interesting case of a bifurcated SM connected with the presence of a unique morphological variation—an accessory SM found during dissection. In the mentioned study, SM originated from the anterior superior iliac spine and then divided into medial and lateral heads of the SM [7]. The lateral head traveled to its site of insertion—the medial aspect of the patella; however, the medial head formed a muscular belly to the vastus medialis and later united with accessory SM, which originated from the inguinal ligament, merged with the medial head of the SM and inserted onto the PA [7] (Figure 2).

This muscle is known for its clinical implication in many procedures. It is a perfect flap donor for a variety of transfers—treatment of groin infections after surgical femoral vessel reconstruction, proximally/distally based flap for abdominal wall, hip repair or knee rigidity treatment, and the reconstruction of large and full-thickness abdominal wall defects or after lymphadenectomy procedures when necrotic areas are covered with flaps to protect the healing process [3,8]. Given the clinical significance of this muscle, a thorough understanding of its anatomy and careful consideration of how, or if, it should be utilized in specific cases is essential.

Due to the fact that the SM forms one of the femoral triangle margins, its anatomical variations could occur during an ultrasound examination of the femoral triangle area performed frequently in case of pain and swelling. Typical causes of swelling in such areas are abscesses, hematomas, adenopathies, aneurysms, inguinal hernias, or thrombophlebitis [9]. Additionally, structures like additional heads/muscles in the femoral triangle area might not only provide confusion but also indicate problems such as restricted movement of the hip and knee joints or induce iatrogenic injury during procedures including femoral artery puncture [7].

### 3.3. Quadriceps Femoris

As previously mentioned, the standard description of the QF that can be found in the anatomical textbooks characterizes this muscle as a group compound of four heads: RF, VM, VL, and VI. All of those heads form a common tendon that attaches to the base of the patella [10,11,12,13,14,15,16].

The first reports about the morphological variability of the QF appeared in 1884 with reports about triceps femoris composed only of the RF, VM, and VL [17]. However, nowadays, anatomists discuss the existence of additional components that make up the multiceps femoris. In a study provided by Olewnik et al. [18], an additional head or heads were present in 68 out of 106 dissected limbs (64.1%).

In the literature, the RF is described as a muscle that originates from the anterior inferior spine of the ilium, the direct tendon, and from the cranial surface of the acetabulum, the indirect tendon [19]. It is widely considered quite a conservative instance. However, a few variabilities were noted:

- According to MacAlister [20], the acetabular origin can be absent, the origin from the anterior inferior iliac spine can be doubled, and acetabular and spinous heads can be continuous.
- Tubbs et al. [19] observed a third head of the RF in 83% of dissected cadavers. An additional head was attached superficially to the tendon of the gluteus minimus and deeply to the iliofemoral ligament [19].
- An additional muscular belly was also noted and named the femoral head of the RF muscle [21]. It originated from the intertrochanteric line, traveled distally, fused with the rectus femoris muscle at approximately its mid-length, and, interestingly, was present bilaterally [21].
- Moore et al. [22] presented an interesting report of three additional muscular heads related to the RF. The more medial traveled from the distal part of the proximal tendon of RF origin and coursed distally to attach to a second, laterally placed accessory head that arose from the deep layer of fascia lata [22]. A most lateral variant head arose from the deep surface of the deep layer of the fascia lata via a broad tendon. Additional heads fused and formed a chiasmatic structure [22].

The VM muscle originates from the intertrochanteric line, medial intermuscular septum, and abductor magnus aponeurosis. This muscle is composed of two components—vastus medialis longus (anteroinferior fibers) and vastus medialis obliqus (horizontal fibers) [18].

The VI originates from the anterior and lateral femoral surfaces and the intermuscular septum. It can be fused to the VM or VL to varying degrees and may also descend below the knee to various lengths [5].

The VL muscle consists of three layers. The superficial layer originates from the greater trochanter lateral surface, and its fibers run downwards to the tendon lamina and further to the QF tendon; the intermediate layer originates from the anterior surface of the greater trochanter and joins the intertrochanteric and gluteus medius ridge; and the deep layer originates one-third proximal to the femur [18]. The VL might be doubled, and additional heads may also appear with attachments to the upper part of the anterior intertrochanteric line, anterior greater trochanter, or the anterior border of the femur; however, the VL muscle is mostly variable at the distal attachment site—base/lateral border of the patella or even lateral epicondyle of the tibia [5].

The distal attachment of the QF also presents certain variability. The tendon might form three laminae that correspond to the muscles of the QF, and the superficial tendon can insert onto the anterior surface of the patella and further onto tibial tuberosity or form suprapatellar tendon via fusion with the VI tendon. All three parts can be united into a common flashy mass or join together and form one tendon that continues over the patella [5]. According to research performed by Olewnik et al. [23], the QF tendon is actually composed of four layers. The authors performed a cadaveric study based on 60 formalin-fixed lower limbs with MRI visualization (Table 1).

These four layers were present in all dissected limbs, and, importantly, during MRI imaging, at first glance, tendons seemed composed of three layers; however, in six participants, four layers were distinguishable on MRI when axial scans were analyzed [23].

Additional bellies of muscles that together create the QF are quite frequently observed morphological variations. The fact that the tendon of the QF is formed of multiple layers may be as clinically important, especially since the QT tendon graft is increasingly used in ACL reconstruction [23]. Presumably, the fact that it is not always possible to distinguish the four layers of the tendon during imaging may be important during pre-operative preparations. However, further studies on these subjects are compulsory in order to draw conclusions.

### 3.4. Tensor of the Vastus Intermedius

Golland et al. [24] were among the very first to report about an additional head of the QF; however, in 2016, Grob et al. [14] published research in which the authors identified, characterized, and named another additional head of the QF—Tensor of the Vastus Intermedius (TVI) [12,14,15]. There is a growing quantity of publications about this relatively recently discovered muscle, which can be found between the VL and VI. In research performed by Grob et al. [14], the muscle belly of the TVI was found in all 26 investigated limbs. Another research accomplished by an example of a previously mentioned study was undertaken by Olewnik et al. [25] and resulted in the presentation of the TVI in 68 out of 106 dissected limbs (64.1%).

**Figure 3:**
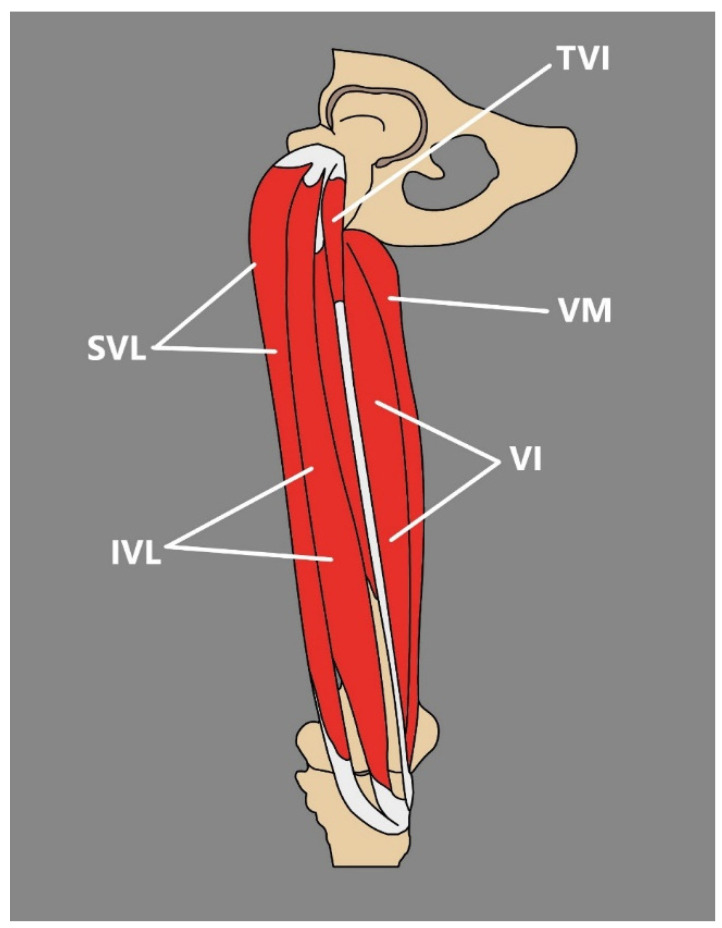
Typical position of the TVI. TVI tensor of the vastus intermedius. SVL: superficial part of the vastus lateralis. IVL: intermediate part of the vastus lateralis. VI: vastus intermedius. VM: vastus medialis.

As already stated, the TVI may occur between the VL and VI muscles. Its muscle belly is usually positioned in the proximal third of the thigh, and it merges into the tendinous structure in the distal third of the thigh; this courses obliquely to its point of insertion to the patella, which occurs at the medial aspect of the patellar base [14,15] (Figure 3).

The absence of studies about the TVI before 2016 might be explained by several reasons, such as the fact that the TVI and VL are located close to one another in the proximal thigh, and both of them are covered by a complex network of nerves and vessels. The TVI is also rarely seen in surgical routine because of its anatomical position [14].

There are several types of classifications of the TVI in the current literature. Grob et al. [14] suggested a classification based on the ability of separation the TVI tendon from the VI and VL muscles. Veeramani et al. [26] describe a congenital classification among a South Indian population. Olewnik et al. [25] provide a categorization of additional heads in which the TVI was noted in 64.1% of dissected limbs (Table 2).

**Table 2:** Types of QF supplementary heads introduced by Olewnik et al. [25].

Type	Description		Occurrence
I	Independent fifth head of QF-TVI, with independent muscle belly that originates from:		
	Subtype IA	The upper level of the anterior surface of the greater trochanter joins the intertrochanteric and gluteus medius ridge; muscle belly runs laterally to the VI, and its tendon passes medially	29.4%
	Subtype IB	The upper level of the anterior surface of the greater trochanter joins the intertrochanteric and gluteus medius ridge; muscle belly runs medial to the VI	14.7%
II	TVI that originates from other muscles:		
	Subtype IIA	From the VL	23.5%
	Subtype IIB	From the VI	4.5%
	Subtype IIC	From the gluteus minimus	2.9%
III	Multiple supplementary heads:		
	Subtype IIIA	Two heads with common tendon: lateral head that originates from the upper level of the greater trochanter’s anterior surface and joins the intertrochanteric and gluteus medius ridge; and medial head, that originates from the femur’s anterior surface just above the VI muscle’s proximal attachment	5.9%
	Subtype IIIB	Two heads with two separate tendons: lateral that originates from the upper level of the greater trochanter’s anterior surface where it joins the intertrochanteric and gluteus medius ridge; medial with origin from the femur’s anteromedial surface just above the VI muscle proximal attachment	14.7%
	Subtype IIIC	Three heads: lateral and intermediate that originate from the VL and form a common tendon, and medial with origin from the upper level of the greater trochanter’s anterior surface and joins the intertrochanteric and gluteus medius ridge	2.9%
	Subtype IIID	Four heads (bifurcated lateral and medial): bifurcated medial, that consists of medial and lateral heads—medial that originates from the femur’s innominate tubercle and lateral from m the inferior level of the greater trochanter’s anterior surface; and bifurcated lateral that consists of medial and lateral heads—medial from the inferior level of the greater trochanter’s anterior surface and from the intermediate part of the VL and lateral that originates from the intermediate part of the VL and from the antero-lateral surface of the shaft of the femur (Figure 4)	1.5%

**Figure 4:**
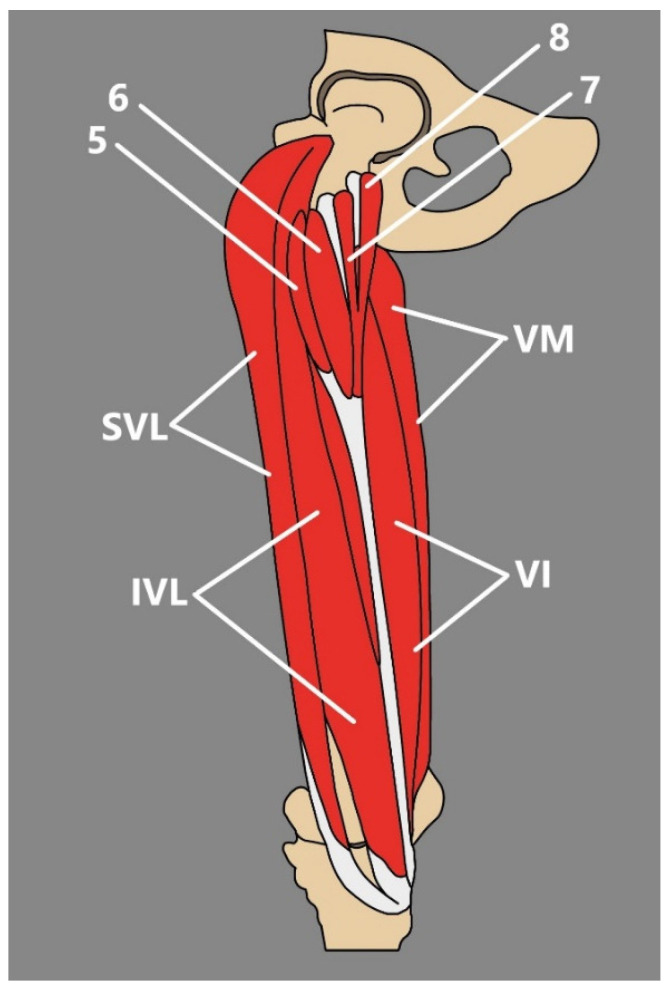
Type IIID of the QF by Olewnik et al. [25]. SVL: superficial part of the vastus lateralis. IVL: intermediate part of the vastus lateralis. VI: vastus intermedius. VM: vastus medialis. 5: fifth head. 6: sixth head. 7: seventh head. 8: eighth head.

Currently, the subject of the occurrence of the TVI muscle in an ultrasound examination is not sufficiently described, which can be understandable, assuming the earlier failure to recognize the TVI. According to Rajasekaran and Mederic [27], the distal tendinous portion of the TVI had previously been observed in a sonographic imagining of the anterior thigh or knee, but it was simply assigned to the VL muscle. The authors propose that the easiest way to identify the TVI during sonographic examination is to use the transverse plain [27].

As a consequence of its anatomical position, the TVI might be a potential sonographic pitfall if unrecognized: as it courses from lateral to medial, it might give the impression of the appearance of focal thickening of the quadriceps tendon and could easily be incorrectly recognized as a region of focal tendinosis, or as a region of hypoechoic tendinosis if it is relatively anisotropic in regard to the under and overlying quadriceps tendon layers [27]. Interestingly, as a consequence of the positioning of the RF and TVI, knowledge about the existence of a TVI might also lead to misdiagnosis when it comes to injury of the RF connected with eccentric contraction of the QF muscle. This type of misdiagnosis was suggested by Ruzik et al. [12], and the reason behind that, presented by the authors, might be the fact that the TVI muscle is capable of undertaking eccentric contraction.

Additionally, because of its position, the TVI might cause difficulties during the USG Doppler examination of the femoral vessels during routine examination of the patency, checking the presence of blood clots or valve condition, by simply obstructing access to those vessels. Following reports that atherosclerotic changes happen to be more advanced in the femoral than in the carotid artery, Kocyigit et al. [28] proposed that an ultrasound examination of the femoral artery could be performed in order to predict the risk of cardiovascular disease. Potentially, the occurrence of the TVI might obstruct access to the femoral vessels during imaging and make examination difficult. Furthermore, the TVI could potentially be misdiagnosed as a soft-tissue tumor, since they might arise from muscle tissue. The TVI could be considered a lipoma or adipocytic tumor or a traumatic or other pseudotumor since the echogenicity of the lesions might progress from hyperechoic to hypoechoic over time [29]. Lastly, there are reports about the occurrence of sarcomas of the medial vastus [30], so the TVI could also be mistaken as one of those, especially if it is positioned more medially, such as in the case presented by Ruzik et al. [13] or type II according to Olewnik et al. [25].

There is a deficiency of sonographic studies embracing the subject of the TVI. This issue should be expanded in the coming years, since anterior knee pain is one of the most commonly reported symptoms in the field of orthopedics [31]. Clinicians should be aware of the existence of the TVI muscle, its function, and the fact that this morphological variation might be a potential sonographic pitfall in order to prevent misdiagnosis.

## 4. Medial Thigh

The medial thigh is typically composed of the gracilis muscle (GM), pectineus muscle (PM), adductor magnus muscle (AM), adductor longus muscle (AL), and adductor brevis muscle (AB).

### 4.1. Gracilis Muscle

The GM is a long and slender muscle that originates from the anterior body of the pubis, inferior pubic ramus, and ischial ramus and inserts onto the medial surface of the proximal tibia via the pes anserinus [32]. This muscle presents little variability. In some cases, the upper fibers of this muscle connect with the fascia lata and fascia of the leg or send a muscular slip that merges with the medial head of the gastrocnemius [5]. An anomalous muscle with two heads was reported—with normal and lower heads that arose from the ramus of the ischium [5].

Over the years, the GM has had numerous clinical applications: facial reanimation, lower-limb trauma, functional transfer to the upper limb, perineal reconstruction or breast reconstruction [33]. Recently, it has been applied in the management of groin wounds after vascular surgery as a free muscle flap, so currently, there is significantly more interest in its extramusculary and intramusculary supply rather than in the morphological variability of its structure [34,35]. Kurtys et al. [35] distinguished three types of intramuscular innervation and also conducted a four-fold classification of extramuscular innervation [34].

Little information is also presented in imaging-oriented literature. However, due to its use as a muscle flap, studies have focused on whether ultrasound is a valuable method of assessment of the functional recovery of transplanted muscle. According to Hou et al. [36], the ratio of the cross-sectional area of the transplanted muscle was highly correlated with muscle function, which can be used to dynamically assess muscle recovery after muscle transplantation, and measurements from B-ultrasound can quantitatively reflect muscle strength following GM transfer.

### 4.2. Pectineus Muscle

The PM is a muscle of the anterior thigh that shows little variation. It is presented as a flat, quadrangular-shaped structure that originates from the superior ramus of the pubis and attaches along the pectineal line from the lesser trochanter to the linea aspera [32]. It may be separated into two layers—superficial and deep; it might be partially/completely attached to the capsule of the hip joint or originate from the adductor brevis [5].

**Figure 5:**
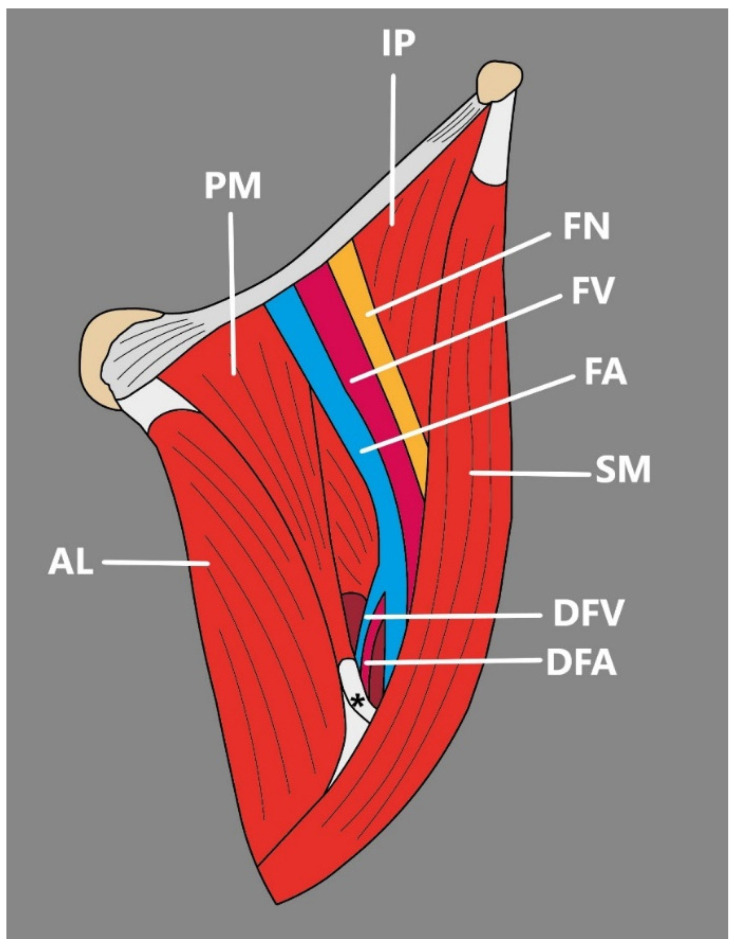
Schematic presentation of the variable pectineus muscle presented by Kim et al. [37]. The tendon of the superficial layer is indicated by an asterisk (*). PM: pectineus muscle. AL: adductor longus muscle. IP: iliopsoas muscle. SM: sartorius muscle. FV: femoral vein. FA: femoral artery. DFA: deep femoral artery. DFV: deep femoral vein. FN: femoral nerve. (*) the tendon of the superficial layer.

Kim et al. [37] were the first to present a variation in which PM formed a hiatus. Bilaterally, the superficial and deep layers of the PM were divided at its distal part, forming a triangular-shaped hiatus between them and the femur shaft [37]. Importantly, on the right side, the deep femoral artery and its first perforating artery passed through the hiatus, and on the left side, the deep femoral artery pierced the hiatus [37] (Figure 5).

Such an anomaly is of great clinical importance, since it can disturb the anteromedial surgical approach aimed at accessing the distal parts of the deep femoral artery when the approach to more proximal vessels is limited. In a patient with pectineal hiatus, when the deep femoral artery passes through the hiatus, retraction of the muscle would be limited, and further forceful retraction might damage the muscle and lead to acute groin pain [37]. In order to prevent this, a pre-operation imaging study should be carried out. Currently, due to the rather scarce number of publications on pectineus—both from an anatomical and clinical point of view, reports about pectineal hiatus in the literature concerning imaging studies are absent. However, clinicians, especially surgeons, should be aware of such structure and its relationship to the deep femoral artery.

### 4.3. Adductor Magnus

The AM muscle is composed of two parts: pubofemoral/adductor minimus and ischiocondylar/hamstring portion [38]. The adductor minimus part arises from the ischiopubic ramus, and the hamstring part arises from the ischial tuberosity [38]. The AM muscle inserts onto the adductor tubercle of the femur and onto the linea aspera [38].

Hildebrand [39] was among the very first to describe the anatomical anomaly of the AM muscle. The author presented a case of a muscular band that arose from the linea aspera right between the insertion site of the AM and with another origin in the short head of the BF muscle that was inserted onto the adductor tubercle [39].

Tubbs et al. [40] presented another case of AM slip that extended from the linea aspera medial lip and inserted into the distal AM tendon as it descended to the insertion site—adductor tubercle. The muscular slip introduced by Tubbs et al. [40] compressed the proximal popliteal vein and led to the proximal popliteal vein aneurysm. Popliteal vein aneurysms are subsequently rare compared to those of the popliteal artery, and additionally, they remain mostly asymptomatic if deep vein thrombosis or pulmonary embolism does not occur [41]. Nevertheless, they can be spotted during ultrasound evaluation.

### 4.4. Adductor Longus

AL originates from the body of the pubis, inferior to the pubic crest and lateral to the pubic symphysis, and inserts onto the middle third of the linea aspera of the femur [32].

AL is considered a muscle of rather constant anatomy. It may be fused with the AM or extend to the knee, which fuses with the AM tendon and makes their attachment inseparable [5]. Kozioł et al. [42] recently presented a rare case of AL anomaly—bilateral accessory heads. The authors observed two additional heads of the right AL, both of which originated at the inferior pubic ramus, close to the pubic tubercle, as well as one of the left AL, which also originated close to the pubic tubercle at the inferior pubic ramus [42]. According to Kozioł et al. [42], the accessory heads of AL were previously reported once by Tuite et al. [43].

Just as in anatomical literature, there is a deficiency of clinical considerations and radiologic images that include additional components of AL. It can be suspected that just as additional heads are presented in other compartments of the human body, such anomalies could mimic pathologies, namely soft-tissue tumors or the Baker’s cyst, often searched during radiologic examination. Moreover, AL forms the posterior wall of the adductor canal, where the femoral artery, femoral vein, and saphenous nerve pass toward the popliteal fossa. It is possible that the additional heads, depending on their morphology and position, could affect structures that pass the adductor canal. Nonetheless, further studies are needed to confirm the presented considerations.

Interest in the AL has grown, since it is the most frequently affected muscle in groin injuries. In a study by Renstrom et al. [44], the AL was injured in 62% of 55 cases of groin injury. Due to this reason, the number of publications on AL imaging continues to grow. According to Pesquer et al. [45], it is important to note the anatomical ultrasound variability of the superficial portion of the AL tendon, which can appear blurred due to the oblique fibers originating from the rectus abdominis and contralateral muscles. Referring to Pasquer et al. [45], anatomical variations on ultrasound can make diagnosis more challenging in case of chronic rearrangements.

### 4.5. Adductor Brevis Muscle

The AB has a narrow origin from the anterior surface of the body of the pubis and the inferior ramus of the pubis and inserts onto the superior half of the medial lip of linea aspera [32]. Little information on AB variability has been introduced to the literature. The muscle can be divided into two or three separate parts to varying degrees, or the muscle may be merged with the AM [5].

Just as in anatomically-oriented literature, the number of publications on the clinical meaning of the AB is rather deficient and usually connected with other adductor muscles. Davis et al. [46] presented a study on proximal adductor tendons and were among the very first to report that AB and AL present intramuscular tendons. Functionally, the intramuscular tendon is responsible for adding additional strength and stability during muscle contraction, which is important, especially among adductors, whereby the musculotendinous junctions are subjected to large mechanical stress [46]. Adductor injury can occur at the proximal tendon or musculotendinous junction. Davis et al. [46] also reported that in all 20 dissected limbs, AB and gracilis were proximally fused. Such findings need to be considered as possible factors that contribute to the pathogenesis and pattern of injuries in adductor-related groin strain [46]. Therefore, awareness of the intramuscular tendon might be important in diagnostic imaging; however, more US or MRI studies on this subject are required.

## 5. Posterior Thigh

According to classical anatomical description, the posterior thigh is composed of the semitendinosus muscle (STM), semimembranosus muscle (SMM), and biceps femoris muscle (BF).

### 5.1. Semitendinosus Muscle

The STM is located in the postero-medial aspect of the thigh, which, together with both heads of biceps femoris and semimembranosus muscle, forms a group called the hamstrings; these share the same function, which is to extend the hip joint, flex the knee and to rotate the knee from side to side at the time of the knee flexion. It typically originates from the superior part of the ischial tuberosity together with the long head of biceps femoris, forms a belly divided by a tendinous inscription into the proximal and distal compartments, which then becomes a long tendon implicated in the formation of the PA [47,48,49]. The STM itself shows little variation [50]; however, the PA is different.

The PA is a structure located in the antero-medial surface of the proximal part of the tibia formed by three conjoined tendons: the semitendinosus tendon (STT), gracilis tendon (GT), and the sartorius tendon (ST). Fusion of those tendons results in the formation of the anserinus plate, which can be divided into a superficial layer formed by the ST and a deep layer formed by the GT and STT [51].

As other tendons are involved in the formation of the PA, the STT manifests an incredible degree of variations, which were included in the morphological classification of the PA anatomy created by Olewnik et al. [51] (Table 3).

**Figure 6:**
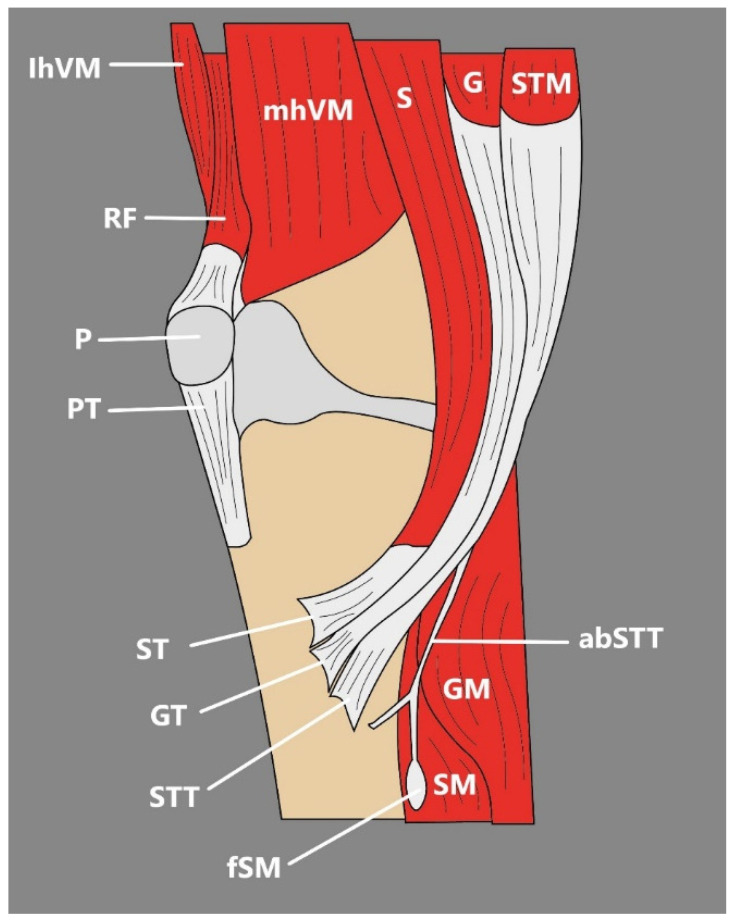
Variation of Pes Anserinus by Olewnik et al. [47]. IhVM: lateral head of the vastus lateralis. mhVM: medial head of the vastus medialis. RF: rectus femoris muscle. P: patella. PT: patellar tendon. S: sartorius muscle. ST: semitendinosus tendon. G: gracilis muscle. GT: gracilis tendon. STM: semitendinosus muscle. GM: gastrocnemius muscle. abSTT: accessory band of the semitendinosus tendon. SM: soleus muscle. fSM: fascia of the soleus muscle.

The above classification was prepared on the basis of 102 lower-limb dissections and takes into consideration not only the differences in the number of accessory bands but also the shape of the insertion and distinguishes short, fan-shaped, and band-shaped types [51]. There are also reports of more unusual accessory STT bands, such as ones with attachment to the deep fascia [48], tibia, and soleus muscle [47] (Figure 6) or PA formed by the GT, ST, STT, its additional bands, and interestingly, the tibial collateral ligament [52].

Recently, Zielinska et al. [53] reported an interesting case of PA variation. The STT was located superiorly to the GT, and they both had distal attachments on the medial side of the tibial tuberosity. The ST created an additional superficial layer, its proximal part lying just below the GT and covering the STT and a small part of the GT. After crossing the STT, it is attached to the crural fascia significantly below the tibial tuberosity [53].

Any sign of morphological variability among the STT is important to notice, since, together with GT, the STT has become a commonly used autograft material for the reconstruction of anterior cruciate ligament (ACL), one of the most frequently performed procedures in the field of rthopedics [47,54,55]. The STT and GT have become common types of implants for ACL reconstruction because the procedure of obtaining these tendons causes fewer complications and demonstrates minor clinical functional impairment [55,56]. However, there are publications about the difficulty connected with grafting tendons from the PA, which assumingly might be connected with significant variability of the STT, GT, or ST; as such, the proper pre-surgery preparation connected with imaging of the PA, its peripheral structures, and their relations might be crucial to achieving concrete therapeutic results [47,55]. Performing imaging before the reconstruction procedure is also important because the tendons present a great variety not only in terms of additional bands but also in terms of length, shape, and thickness, which directly influences the decision whether to use STT alone or to also use GT [54]. There were many propositions of imaging techniques in the pre-surgery preparation, such as MRI, arthroscopy, or 3D-CT, although the use of ultrasound seems to be justified, since it is more commonly used in the evaluation of the soft-tissue structures around the knee [54]. According to Zhong et al. [55], the consistency between anatomic and ultrasonic measurements of the PA implies the US is a significant device in STT, GA, and ST imaging.

Interestingly, the occurrence of additional bands of the STT is not notably explored in imaging studies, which seems to be bothering, not only because of its potential implications during the procedure of harvesting the STT or GT for ACL reconstruction but also since their locations and forms are quite variable, their occurrence spotted by an unaware clinician may be mistaken with the pathology of the PA [47]. Such a situation is possible, especially since US examination is a frequently used device during imaging of the structures around the knee and during detecting pathologies of the PA, including anserine bursitis, pes anserinus cysts, and pes anserinus tendonitis [55].

### 5.2. Semimembranosus Muscle

The SMM is another example of a variable hamstring muscle. It originates as a membranous tendon from the lateral surface of the ischial tuberosity, forms a muscular belly, courses downwards, and forms a distal tendon that divides into five units [57,58] (Table 4).

The SMM muscle can be split along its length, arise proximally from the coccyx or sacrotuberous ligament, connect with the adductor magnus or femur through tendinous slips, or occur as partly fused with the ST [5].

Interestingly, a congenital bilateral absence of the SMM muscle was also reported [5,59]. Such a situation was described, among others, by Sussmann [59], who presented a case of complete deficiency of both tendon and belly of the SMM during a MRI examination of a 42-year-old knee. This resulted in a fat-filled SMs muscle fossa and unconventional morphological changes of the ST and biceps femoris, which might compensate for the absent SMM [59]. According to Sussamann [59], such an absence might predispose the patient to medial meniscal posterior horn injury or posteromedial capsule injury. Importantly, the absence of the SMM might affect the results of ACL reconstruction, since ST or GT are frequently harvested in this procedure [54]. Hamstring strength deficits were reported after grafts for ACL reconstructions, so losses due to harvest might not be fully compensated, especially when the SMM muscle is absent [59]. Although surprising, the lack of the SMM muscle can be easily spotted during pre-operative US examination, based on the simple absence of the characteristic hyperechoic structure of the SMM [60]. If such an absence is presented, grafts should be taken from areas other than the hamstring tendons [59].

The accessory SMM muscle was found to arise from the distal part of the SMM, traverse the popliteal fossa laterally to the SMM and ST, but medially to the biceps femoris, bypass the PA, and insert onto the proximal aspect of the medial head of gastrocnemius [61]. Such a structure could be easily misinterpreted as a soft-tissue lesion during not only ultrasound examination but also MRI imaging, so determination of its origin and insertion is advised in order to prevent potential misdiagnosis [61].

Interestingly, the SMM is also variable in the number of its distal insertions, since it can present an additional sixth insertion: the tendinous slip onto the medial third of the lateral meniscus [62]. According to Kim et al. [62], just like other distal attachments of the SMM, it contributes to the medial stability of the knee and was present in 43.2% of studied cases. Importantly, it can mimic a tear of the lateral meniscus during both US and MRI imaging, so it is important to be aware of it in order to prevent unnecessary diagnostic procedures [62].

### 5.3. Biceps Femoris

The BF muscle is composed of two heads: a long head arising together with the STT muscle from the ischial tuberosity and from the sacrotuberous ligament, and a short head arising from the lateral lip of linea aspera, lateral intermuscular septum, and the supracondylar line [63]. Those heads then fuse together and insert into the head of the fibula, crural fascia, and proximal tibia [63].

The BF muscle is not perceived as variable; however, there are a few reports about the unique existence of the third head. Tsunekawa et al. [64] spotted such instances during dissection, where the third head had two origins—one on the femur and another from the gluteus maximus tendon. The third head tendon joined the long and short heads of the BF muscle. Previous mentions of the third head suggested that it may originate from the pointed pelvis, femur, gluteus maximus, bursa trochanterica musculi glutei maximi, gluteus medius, piriformis and adductor magnus [64]. Arakawa et al. [65] also found an additional muscular component of the long head of the BF muscle, which was fused with the STT muscle.

**Figure 7:**
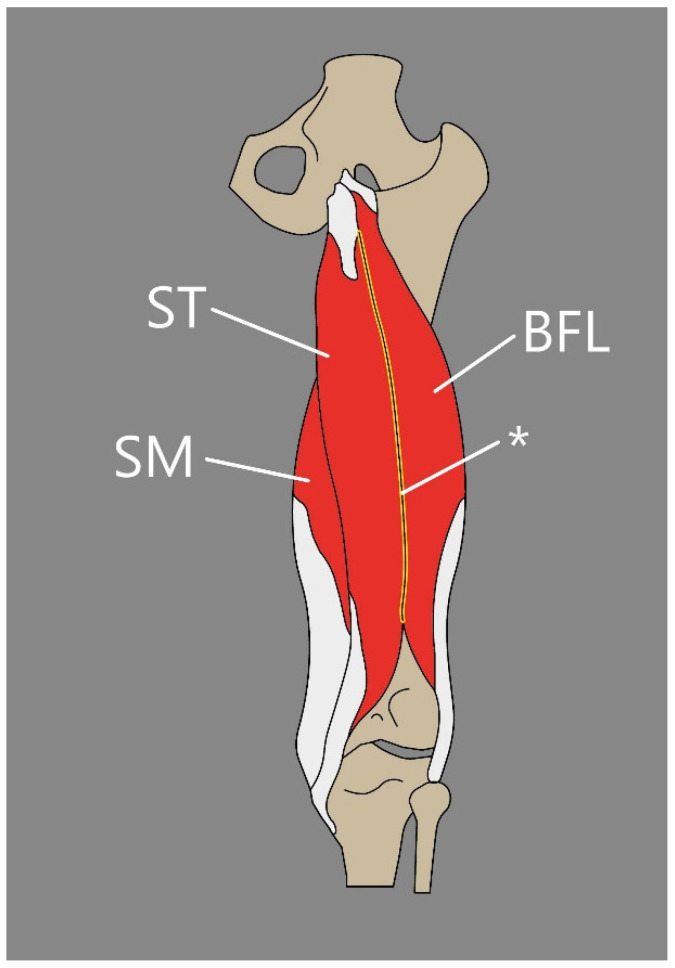
Schematic presentation of a fusion between semitendinosus muscle and long head of biceps femoris. ST: semitendinosus muscle. BFL: long head of biceps femoris. SM: semimembranosus muscle. (*) fusion between BFL and SM.

Another interesting instance of the BF variability is also the rarely noted fusion between the long head of BF and SMM (Figure 7). Schmuter et al. [66] presented a case study indicating a bilateral fusion from the common head at their origin—ischial tuberosity. Additionally, during dissection, a muscular slip that arose from the lower border of the gluteus maximus and blended with the long head fibers was found [66].

Such additional muscular components or fusions could appear clinically as the reason for sciatic nerve compression since bellies of BF and SMM are closely associated with the sciatic nerve [66]. It can be suspected that such neuropathy could be diagnosed during ultrasound examination; however, this may not be easy, since those variabilities are quite unique and not widely known among clinicians. It is also important to note that the additional head of the BF muscle could be easily mistaken as a soft-tissue tumor during US examination, for example, during the search for the cause of sciatic nerve compression.

### 5.4. Tensor Fasciae Suralis

Tensor Fasciae Suralis (TFS) is an extremely rare instance with an unknown prevalence in humans, also known as an ischioaponeuroticus. Kelch [67] and Gruber [68] were among the very first to present a description of this muscle, which was further explored by Schaeffer [69] and Bergman et al. [5].

**Figure 8:**
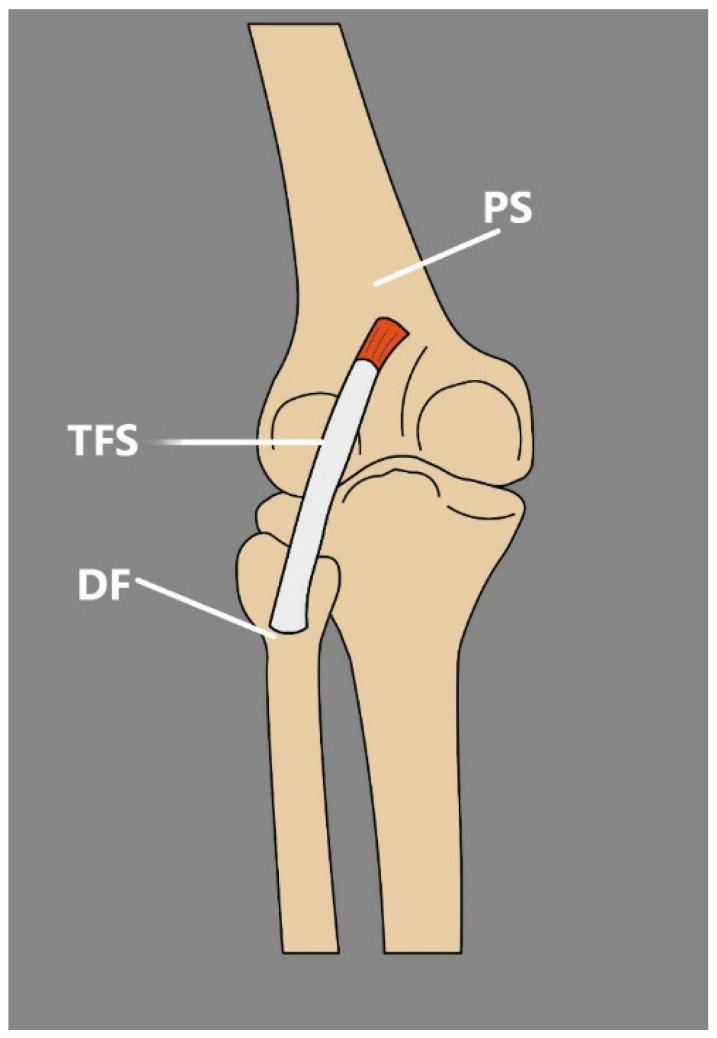
Schematic presentation of the tensor fasciae suralis reported by Olewnik et al. [2]. TFS: tensor fascia suralis. PS: popliteal surface of the femur. DF: deep fascia.

The TFS is usually described as a muscle that arises either from the semitendinosus or long head of biceps femoris, or both of them; it forms a long and thin tendon positioned superficially beside the popliteal fossa and then attaches to the sural fascia or gastrocnemius or superficial aspect of the Achilles tendon [70,71]. However, the muscle has some interesting variations:

- Olewnik et al. [2] reported an exceptional possible variant that was located deeply under the biceps femoris muscle with an origin on the popliteal surface of the femur and insertion into the deep fascia of the leg (Figure 8).
- Bale et al. [71] describe an incidence of the TFS that occurred bilaterally and, interestingly, was not the only accessory muscle presented during dissection. Recently, Bale et al. [72] proposed a classification system for the TFS variation (Table 5).

Since the TFS is not commonly observed, no studies about this muscle and its function have been carried out, and in current literature, it is only speculated that TFS might assist in the leg flexion and the placement of the fascia of the posterior leg [73].

Although the TFS is uncommon, it is clinically significant. Montet et al. [74] were the first to present a report in which the appearance of the TFS was introduced as a cause of popliteal swelling, which provided the foremost images from MRI and ultrasound examination. More recently, Tsifountoudis et al. [70] offered a case report in which an 18-year-old male presented with acute pain in the right popliteal fossa. Further physical examination disclosed swelling and tenderness connected with a palpable mass that occurred in the popliteal region and was covered by healthy, typical skin. MRI and a US examination were commissioned [70].

During the ultrasound imaging, both axial and longitudinal, a fusiform-shaped structure arising from the semitendinosus muscle was noted, which extended into the popliteal fossa and merged into the medial head of the gastrocnemius. The observed structure had characteristic features that can be noticed during US examination and was properly recognized as the TFS muscle based on its anatomical positioning. Ultrasound imaging also revealed hypoechoic fluid collection, which indicated grade I muscle strain injury.

Conducting an imaging study is quite significant in case of TFS injury, since it can be easily misdiagnosed with other causes of popliteal swelling such as soft-tissue tumors, aneurysms of popliteal artery, abscesses, or Baker’s cysts [2,70,75,76]. Additionally, since the TFS is positioned close to the neurovascular bundle, it can compress not only the popliteal vein but also the popliteal artery and surrounding the sciatic, tibialis, or sural nerves [77,78,79]. Comparable circumstances might lead to confusion about the appearance of the TFS with soft-tissue tumors or aneurysms of the popliteal artery [2,74]. If the TFS is present in an anatomical position, such as in the previously mentioned study by Olewnik et al. [2], its rupture might be the cause of femoris tendon rupture, and injury of the TFS might lead to the trauma of the fibular collateral ligament.

Even though the occurrence of the TFS is infrequent and remains unclear among humans, clinicians should be familiar with this structure. It can cause some confusion when it comes to the proper diagnosis of popliteal swelling and because a lack of knowledge about this structure might lead to unnecessary actions, like investigation and intervention. Ultrasound examination seems to be a reasonable choice in the situation of visualizing the TFS muscle belly and tendon, since it can be easily observed in its entire length during imaging.

## 6. Conclusions

Our understanding of human anatomy is constantly evolving, as demonstrated by the uncountable morphological variations of numerous structures that make up the body. The muscles of the thigh are no exception in this matter. Additional heads, accessory muscles, accessory bands, muscular fusions, and tendinous slips are all structures that can be found in anatomy and are not always asymptomatic. Awareness of such instances is clinically relevant, especially in imaging and surgical studies, since they can easily be mistaken for other soft-tissue structures, which can lead to misdiagnosis and incorrect treatment.

## Figures and Tables

**Table 1:** Layers of the QF tendon distinguished by Olewnik et al. [23].

Layer	Components
Superficial (first) layer	RF tendon and fascia
Middle (second) layer	Superficial part of the VL and VM tendon
Middle-deep (third) layer	Intermediate part of the VL
Deep (fourth) layer	VI tendon

**Table 3:** Classification of PA anatomy by Olewnik et al. [51].

Type	Description	Occurence
1-1-1	Monotendinous ST, GT, STT	52.9%
1-1-2	Monotendinous ST, GT, but with an additional band from STT	31.4%
1-1-3	Monotendinous ST, GT, and two additional bands from STT	8.8%
1-2-3	Monotendinous ST, one additional band from GT, and two additional bands from STT	1%
2-1-2	One additional band from ST, monotendinous GT, and one additional band from STT	2%
2-2-3	One additional band from GT and ST and two additional bands from STT	3.9%

**Table 4:** Units of the semimembranosus distal tendon.

Number	Insertion
I	The posteromedial aspect of the tibia deeply to the medial collateral ligament
II	Direct insertion onto tibia
III	Oblique popliteal ligament
IV	Posterior oblique popliteal ligament
V	Popliteus aponeurosis

**Table 5:** Classification of the tensor fascia suralis.

Type		Description	Number of Cases in Study by Bale et al. [72]
I	A	One-headed muscle; origin from the long head of the biceps femoris	16
	B	One-headed muscle; origin from the semitendinous muscle	13
	C	One-headed muscle; origin from the short head of the biceps femoris	2
	D	One-headed muscle; origin from the semimembranosus muscle	0
II		Two-headed muscle; medial head arises from the semitendinosus muscle, lateral head arises from the long head of the biceps femoris muscle	4
III		Other variations	3
